# Primary Multidrug-Resistant *Mycobacterium tuberculosis* in 2 Regions, Eastern Siberia, Russian Federation

**DOI:** 10.3201/eid1910.121108

**Published:** 2013-10

**Authors:** Svetlana Zhdanova, Scott K. Heysell, Oleg Ogarkov, Galina Boyarinova, Galina Alexeeva, Suporn Pholwat, Elena Zorkaltseva, Eric R. Houpt, Eugeniy Savilov

**Affiliations:** Russian Academy of Medical Sciences, Irkutsk, Russian Federation (S. Zhdanova, O. Ogarkov, E. Savilov);; University of Virginia, Charlottesville, Virginia, USA (S.K. Heysell, G. Boyarinova, S. Pholwat, E.R. Houpt);; Regional TB-Prevention Dispensary, Irkutsk (O. Ogarkov, E. Zorkaltseva);; Research Practice Center for Phthisiatry, Yakutsk, Russian Federation (G. Alexeeva);; State Medical Continuing Education Academy, Irkutsk (E. Zorkaltseva, E. Savilov)

**Keywords:** tuberculosis, multidrug-resistant tuberculosis, MDR TB, HIV, Siberia, Russian Federation, genotype, mycobacterial interspersed repetitive unit, MIRU-VNTR, *pncA*, pyrazinamide, tuberculosis and other mycobacteria

## Abstract

Of 235 *Mycobacterium tuberculosis* isolates from patients who had not received tuberculosis treatment in the Irkutsk oblast and the Sakha Republic (Yakutia), eastern Siberia, 61 (26%) were multidrug resistant. A novel strain, S 256, clustered among these isolates and carried *eis*-related kanamycin resistance, indicating a need for locally informed diagnosis and treatment strategies.

In 2010, tuberculosis (TB) prevalence in the Russian Federation was 136 cases per 100,000 population; the estimated proportion of multidrug resistance, defined as resistance to isoniazid and rifampin in the absence of prior treatment (primary MDR TB), was 18% (*1*). However, at the subnational level, primary MDR TB might be highly variable; in oblasts or republics with continuous surveillance data, drug resistance varies from 5.4% to 28.3% (*2*). These data are predominantly from the western half of the country and do not include eastern Siberia.

In 2009, in the Irkutsk oblast in eastern Siberia, TB prevalence was 373 cases per 100,000 population and HIV prevalence was among the highest in the Russian Federation (*3*,*4*). In contrast, in the sparsely populated neighboring Sakha Republic (Yakutia), TB prevalence was lower (188 cases/100,000 population) and HIV was thought to be scarce (*4*). Molecular typing has found that more than half of the *Mycobacterium tuberculosis* isolates from the Russian Federation are the Beijing genotype, a pandemic lineage associated with MDR phenotype and characteristic drug-resistance mutations; prevalence of this genotype in Irkutsk is high (*5*,*6*). However, such investigation has not been performed in Yakutia. Given the distinct sociocultural patterns between Irkutsk and Yakutia, we hypothesized that the molecular epidemiology and drug-resistance patterns of *M. tuberculosis* from patients with primary MDR TB would be regionally distinct.

## The Study

From November 2008 through May 2010, *M. tuberculosis* isolates were cultured during routine care of adults >18 years of age with primary TB and no history of treatment. The patients were from 2 regional referral centers, the Irkutsk Regional TB-Prevention Dispensary and the Research Practice Center for Phthisiatry (Yakutia); the study was approved by the institutional review boards at the University of Virginia and Irkutsk State Medical University.

Initial pretreatment isolates were grown on Lowenstein-Jensen agar slants and identified to species in accordance with World Health Organization recommendations. Drug susceptibility was tested by absolute concentration method on agar slants; drugs tested were rifampin (critical concentration 40 µg/mL), isoniazid (1 µg/mL and 10 µg/mL), ethambutol (2 µg/mL), streptomycin (10 µg/mL), ethionamide (30 µg/mL), and kanamycin (30 µg/mL). Susceptibility to a fluoroquinolone and pyrazinamide was not routinely tested. DNA extraction was performed on all isolates, followed by 12-loci mycobacterial interspersed repetitive unit–variable number tandem repeat (MIRU-VNTR) analysis (*7*) and further lineage definition by region of difference deletions, or for Ural strains as described (*5*). Phylogenetic tree construction was based on the MIRUVNTR*plus* database (*8*), and VNTR international type numbers were confirmed on the SITVIT database (*9*). DNA from MDR isolates was amplified and sequenced for the known drug-resistance determining regions *katG*, *inhA*, *rpoB*, *embB*, *gyrA*, *rrs*, and *eis* by using methods described by the Centers for Disease Control and Prevention (*10*). For *pncA*, the entire open reading frame and upstream promoter region were amplified. Sequences were compared with published sequences for *M. tuberculosis* H37Rv by using GeneDoc version 2.7.0.

Among 235 patients with primary TB (130 from Yakutia, 105 from Irkutsk), isoniazid monoresistance was found in isolates from 16 (12%) from Yakutia and 19 (18%) from Irkutsk (p = 0.27). Multidrug resistance was found for 61 patients (36 [28%] from Yakutia and 25 [24%] from Irkutsk) (p = 0.55). Mean age (± SD) for these 61 patients was 33 (± 12) years, 40 (66%) were male, and these characteristics did not differ significantly between patients from Irkutsk and from Yakutia. However, no HIV-infected patients were identified from Yakutia compared with 11 (44% with MDR TB) from Irkutsk (p<0.001). Twelve MDR TB patients from Irktutsk died (outcome unknown for the other 13 patients), including all with HIV, compared with 4 (11%) from Yakutia who died (p = 0.002). Follow-up varied and was limited mostly to inpatients.

Among all 235 patients with primary TB, strains of the Beijing family were significantly more common among those from Irkutsk (70 [67%]) than from Yakutia (40 [31%]) (p<0.001). However, strains found in Yakutia (S 256 [11%], T 8 [7%], and Ural 171 [5%]) were not found in Irkutsk (Table 1). The cluster of S 256 (MIRU profile 233325153325) was the most common among primary MDR TB isolates from Yakutia and was fully 86% MDR (Table 1; Technical Appendix).

**Table 1:** *Mycobacterium tuberculosis* genotype, by region, eastern Siberia, Russian Federation*

MIRU-VNTR 12	Family/ MIT	Irkutsk		p value		Yakutia		p value
		No. (%) total, n = 105	No. (%) MDR, n = 25			No. (%) total, n = 130	No. (%) MDR, n = 36	
223325153533	Beijing 16	32 (31)	7 (28)	<0.001		12 (9)	1 (3)	0.006
223325173533	Beijing 17	13 (12)	6 (24)	0.27		10 (8)	7 (19)	0.76
233325153325	S 256	0	0	<0.001		14 (11)	12 (33)	0.001
223125153324	T 8	0	0	NA		9 (7)	0	0.005
227225113223	Ural 171	0	0	NA		6 (5)	0	0.03
223325153433	Beijing 592	1 (1)	0	NA		4 (3)	0	0.38

*MIRU-VNTR, mycobacterial interspersed repetitive unit–variable number tandem repeat (original 12-loci profile). Included genotypes found in >5 isolates only; MIT, MIRU–VNTR international type; MDR, multidrug-resistant tuberculosis (conventional resistance to isoniazid and rifampin); NA, not applicable. Significance determined by χ^2^ analysis with Yates correction or Fisher exact test when appropriate.

Among isolates from patients with primary MDR TB, 51 (84%) were available for DNA sequencing: 27 from Yakutia and 24 from Irkutsk (Table 2). Among isoniazid-resistant isolates, the mutation in codon 315 of *katG* was present in 91%. Among rifampin-resistant isolates, mutations in the resistance-determining region of *rpoB* (codons 511–533) were present in only 79%. The *pncA* mutation was common across genotypes from both sites, occurring in 62% of isolates amplified. Notably, both isolates with mutation in *eis* from Yakutia occurred in MDR strains with the S 256 genotype and without *rrs* mutation.

**Table 2:** Resistance mutations in *Mycobacterium tuberculosis* from 51 patients from Irkutsk and Yakutia, Russian Federation*

Drug, locus	Mutation, no. (% total)		Drug resistance, no. (% with mutation)
Amino acid change	Nucleotide change
Isoniazid			
* katG*	Ser315Thr 28 (55)		25 (89)
	Ser315Thr/Trp321Cys 4 (7)		4 (100)
	Trp321Cys 1 (2)		1 (100)
	Thr322Ala 1 (2)		0
	No mutation 8 (16)		3 (38)
	No amplification 9 (18)		8 (89)
* inhA*		C(−15)T 3 (6)	3 (100)
		T(−8)A 1 (2)	1 (100)
		G (−13)T 1 (2)	1 (100)
		No mutation 41 (80)	32 (78)
		No amplification 4 (7)	4 (100)
Rifampin, *rpoB*	Ser531Leu 19 (37)		15 (79)
	Ser531Leu/Thr481Ala 1 (2)		0
	Ser 531Leu/Thr480Ile 2 (4)		2 (100)
	Ser531Tryp/Val456Gly 1 (2)		1 (100)
	Gln513Lys 2 (4)		2 (100)
	Leu533Pro 1 (2)		1 (100)
	His516Tyr 1 (2)		1 (100)
	Leu511Pro 1 (2)		0
	No mutation 22 (43)		6 (27)
	No amplification 1 (2)		0
Fluoroquinolones, *gyrA*			Not performed
	Ser95Thr 46 (90)†		
	Asp94Gly 1 (2)		
	Asp94Ala 1 (2)		
	Ala90Val 1 (2)		
	No amplification 2 (4)		
Ethambutol, *embB*	Asp354Ala 3 (6)		1 (33)
	Asp354Ala/Gly406Asp 1 (2)		0
	Met306Val 3 (6)		3 (100)
	Met306Ile 3 (6)		3 (100)
	Gly406Ser 3 (6)		2 (67)
	Gly406Ala 2 (4)		0
	Gly406Cyst 1 (2)		1 (100)
	No mutation 25 (49)		9 (36)
	No amplification 10 (20)		3 (30)
			Not performed
Pyrazinamide, *pncA*‡	Gly113Phe 3 (6)§	G338T and C96T	
	Leu19Arg 2 (4)§	T56G	
	Gly113Phe/Arg121 Leu 1 (2)§	G338T and C96T/ G362T	
	Arg121Leu 1(2)§	G362T	
	Gln10Pro 1 (2)	A29C	
	Val7Gly 1 (2)	T20G /G481C	
	Ala161Pro/ Val155Ala 1 (2)§	G203A	
	His137Asp/ Frameshift 1 (2)§	T464C/ insertion C480	
	Tryp68Stop 1 (2)§	C409G	
	Frameshift 1 (2)§	deletionG5	
	No mutation 8 (14)		
	No amplification 30 (59)		
Kanamycin			
* rrs*		A1401G 4 (57)	3 (75)
		C1443G 3 (43)	1 (33)
		No mutation 35	12 (34)
		No amplification 9	3 (33)
* eis*		G(−10)A 4 (44)	2 (50)¶
		C(−14)T 1 (11)	1 (100)¶
		C(−15)G 2 (22)	0
		C(−14)G 1 (11)	0
		C(−12)T 1 (11)	0
		No mutation 36	11
		No amplification 6	3 (50)

*Blank cells indicate not applicable. All *inhA* mutations were associated with a Ser315Thr mutation in *katG* except for 1 isolate in which *katG* did not amplify.  †Previously demonstrated not to be associated with phenotypic resistance. ‡Excluding 25 silent *pncA* mutations (Ser32Ser most common, n = 14). §Mutations in *pncA* not previously reported. Conventional susceptibility testing was unavailable for pyrazinamide and the fluoroquinolones. ¶For all 3 mutations of *eis* associated with kanamycin resistance, *rrs* was wild type.

## Conclusions

In eastern Siberia, >25% of primary TB was MDR, equivalent to the highest proportion reported from the Russian Federation (*2*). However, regionally specific genotypic patterns and resistance mutations were identified. As expected, in Irkutsk primary MDR TB was driven by strains of Beijing lineage (*5*,*6*). Yet in the more geographically isolated population of Yakutia, a strain previously unidentified in the Russian Federation, S 256, had a MIRU profile recently found among Canadian Aboriginal populations (*11*). In Yakutia, S 256 was highly drug resistant and was the most common genotype among patients with primary MDR TB.

Although *rpoB* mutations were found in only 79% of rifampin-resistant isolates, these findings are consistent with those in a recent report from Novosibirsk oblast, which similarly included non-Beijing and S-family strains and found a sensitivity of only 63% for the *rpoB* mutation (*12*). Lack of phenotypic correlation can result from alternate mechanisms of resistance or imperfect conventional susceptibilities in Lowenstein-Jensen medium or from use of old drug stock. Such discrepancy necessitates urgent clarification because substitution of conventional susceptibility testing with molecular probe–based methods such as GeneXpert MTB/RIF (Cepheid, CA, USA) has been strongly advocated but would lead to dramatically different results and treatment regimens (*13*). Of note, isolates of the S 256 strain accounted for a proportion of the cases in which mutation in the promoter region of *eis* was associated with kanamycin resistance, but *rrs* was wild type. Commercial assays have focused on the *rrs* locus, which has greater sensitivity for amikacin, as the sole target for the class of injectable agents (*14*), yet in Eastern Siberia, the injectable agent available is kanamycin. Furthermore, we found a range of reported and unreported mutations across the entire *pncA* gene; most were point mutations resulting in amino acid substitution, but some strains had mutations that resulted in deletion or frameshift. Phenotypic methods and assays of functional pyrazinamidase activity should be performed in this region because results might have major implications for novel MDR TB drugs that work best with pyrazinamide (*15*).

Study limitations include selection bias of isolates from passive surveillance. We were unable to obtain detailed clinical information about all patients with primary TB, thus preventing adequate comparison of nongenotypic risk factors for MDR TB or establishment of definitive epidemiologic links among clustered isolates. Furthermore, lack of conventional fluoroquinolone or pyrazinamide susceptibility testing limited comparison with *gyrA* and *pncA* mutations, respectively. Despite these limitations, this work characterizes severe isoniazid monoresistant and MDR TB in eastern Siberia among patients with no history of TB treatment. The regionally distinct phylogenetic patterns and certain drug-resistance mutations necessitate careful application of novel diagnostics and empiric therapeutic strategies.

Technical Appendix Phylogenetic trees of *Mycobacterium tuberculosis* from patients with primary tuberculosis, Yakutia and Irkutsk, Russian Federation.
