# Supplementary material for: Primary Multidrug-Resistant Mycobacterium tuberculosis in 2 Regions, Eastern Siberia, Russian Federation
Source: Emerg Infect Dis. 2013 Oct;19(10):1649–52. doi: 10.3201/eid1910.121108 (PMC3810730; doi:10.3201/eid1910.121108)
Supplement: Technical Appendix — Phylogenetic trees of Mycobacterium tuberculosis from patients with primary tuberculosis, Yakutia and Irkutsk, Russian Federation. [file 12-1108-Techapp-s1.pdf]

Article DOI: http://dx.doi.org/10.3201/eid1910.121108

# Primary Multidrug-Resistant *Mycobacterium tuberculosis* in 2 Regions, Eastern Siberia

## Technical Appendix

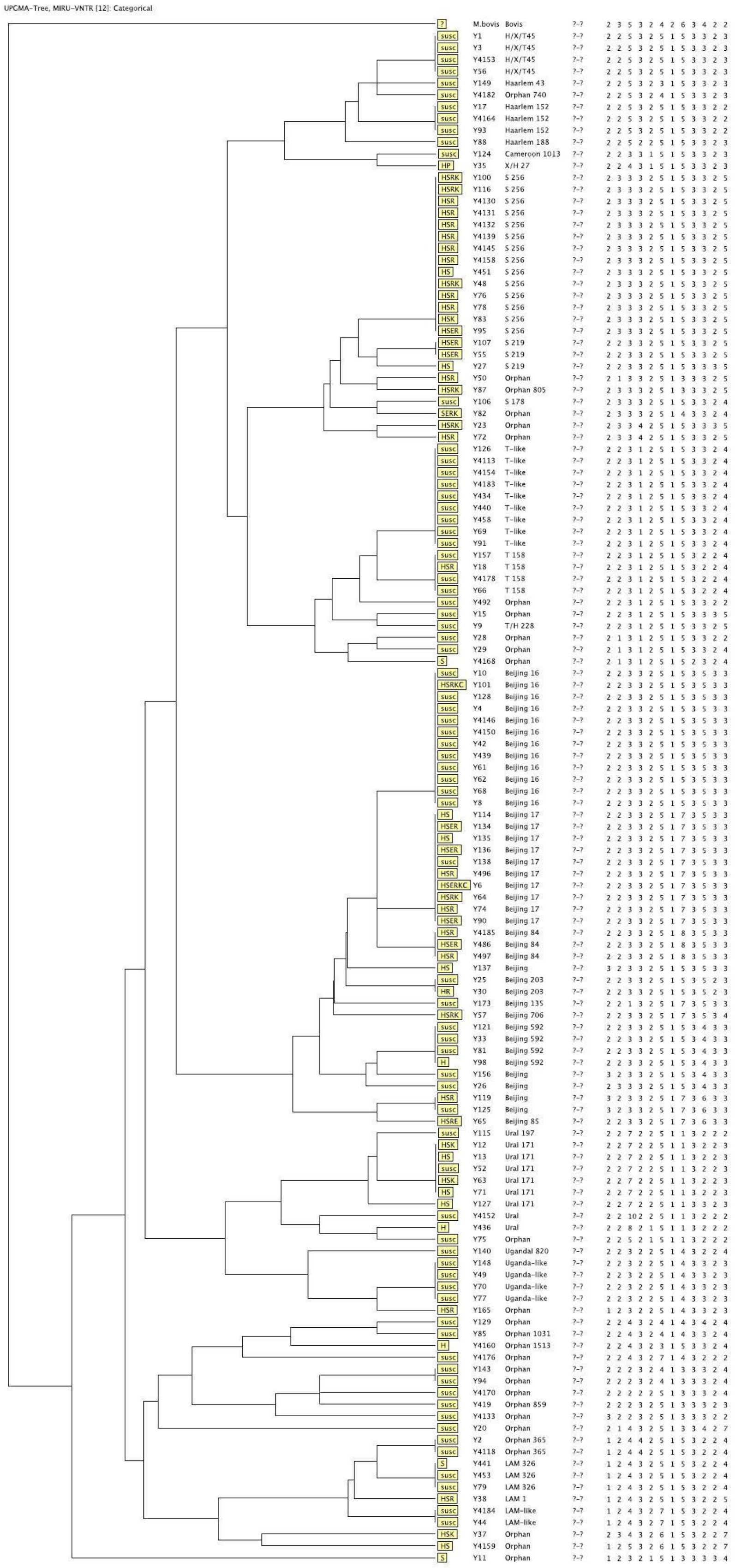


Technical Appendix Figure 1. Phylogenetic tree of *Mycobacterium tuberculosis* from 130 patients with primary tuberculosis, Yakutia, Russian Federation, determined by UPGMA (unweighted pair group method using arithmetic averages) tree by 12-loci mycobacterial interspersed repetitive unit–variable number tandem repeats (MIRU-VNTR). Yellow squares indicate drug resistance; H, isoniazid; S,

streptomycin; E, ethambutol; R, rifampin; K, kanamycin; C, capreomycin; susc, pansusceptible. Strain numbers are followed by lineage, VNTR international type number (if existing), and MIRU profile.

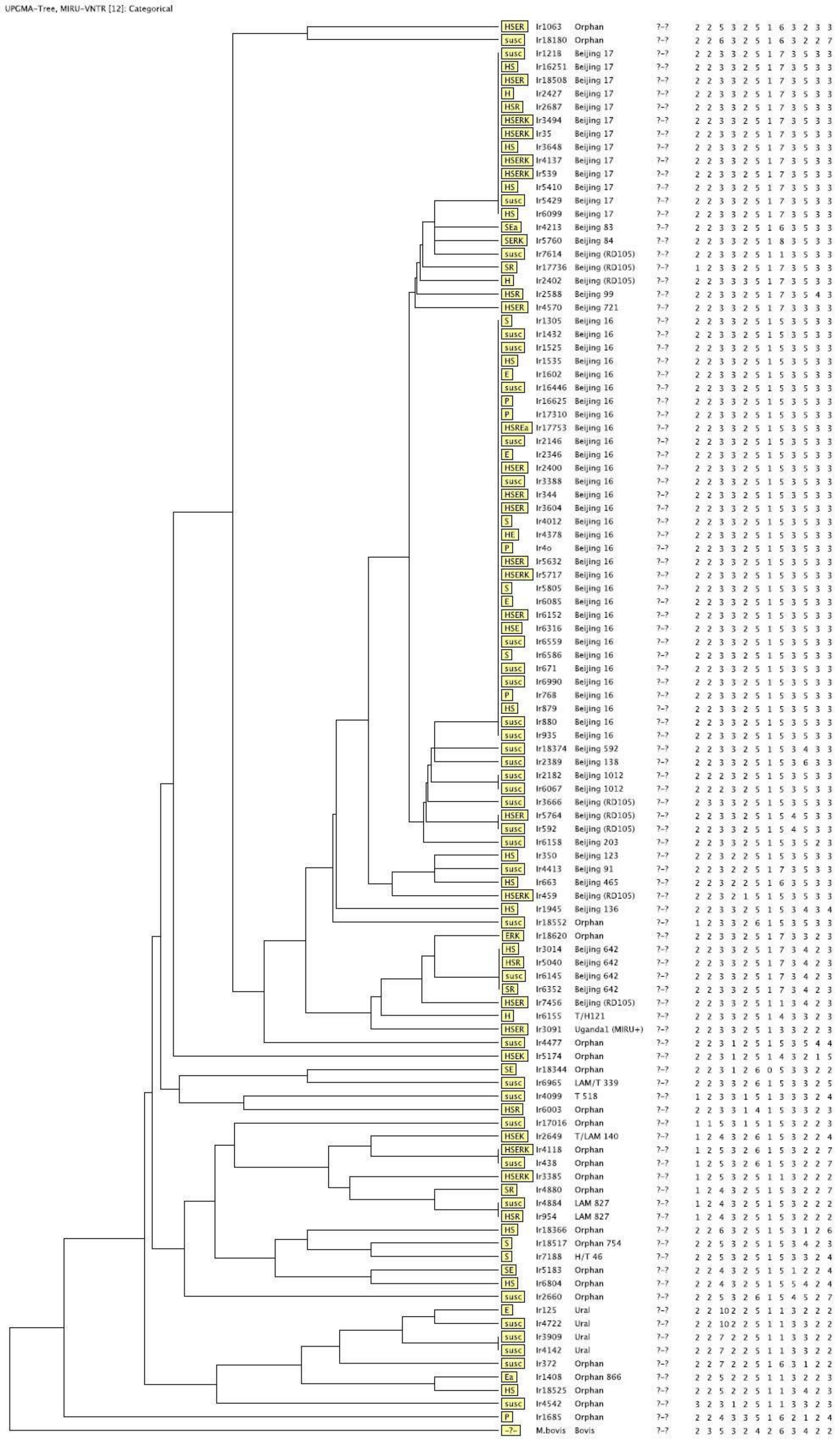


Technical Appendix Figure 2. Phylogenetic tree of *Mycobacterium tuberculosis* from 105 patients with primary tuberculosis, Irkutsk, Russian Federation, determined by UPGMA (unweighted pair group method using arithmetic averages) tree by 12-loci mycobacterial interspersed repetitive unit–variable number tandem repeats (MIRU-VNTR). Yellow squares indicate drug resistance; H, isoniazid; S, streptomycin; E, ethambutol; R, rifampin; K, kanamycin; C, capreomycin; susc, pansusceptible. Strain numbers are followed by lineage, VNTR international type number (if existing), and MIRU profile.
